# Case of Colica Pictonum

**Published:** 1811-07

**Authors:** Robert Harrup

**Affiliations:** Chobham


*To the Editors of the Medical and Physical Journal.*

*Inquiry for a Remedy in cases of Ringworm.*

GENTLEMEN,

I am, Gentlemen,

Your humble Servant,

PHILANTHROPOS.

---

*To the Editors of the Medical and Physical Journal.*

GENTLEMEN,

IF you think the following obstinate and long continued case of Colica Pictonum of sufficient interest, have the goodness to insert it in the next number of the Medical and Physical Journal.

I am, Gentlemen,
Your obedient humble Servant,
ROBERT HARRUP.

*Chobham, March* 8, 1811.

William

William Drew, a labourer in husbandry, about thirty years of age, of a slender make, but generally healthy, applied to me for a pain of his bowels, on the 9th of June, 1810, for which he took a purge of jalap and calomel. Three days after he again applied, said the physic had operated well, but he felt his stomach rather disordered.

Considering this last complaint to arise from acidity, he took a mixture composed of natron, magnesia, and mint water. A few days afterwards, I was sent for to him; he complained much of the pain in the umbilical region, such as was felt when he first applied, but now more severe, and which, he now acknowledged, he had experienced, more or less, for two months past. Tongue furred, pulse 100 and feeble, urine high coloured and passed with difficulty, considerable thirst, no appetite, frequent retching, sometimes bringing up from the stomach a green acid fluid, bowels obstinately constipated, and the stools, when procured, very dark coloured. To these symptoms may be added, giddiness when in an erect posture, great restlessness, no sleep, and the skin of a light yellow colour, which increased in a slight degree as the disease advanced, without any yellow tinge of the eye.

The bowels were attempted to be kept open with the infus. sennæ and magnes. sulphat., glysters, and an occasional pill of a few grains of calomel. Stools were, however, procured with much difficulty, and relieved him in a very trifling degree.

Blisters applied to the scrobiculus cordis and abdomen afforded a temporary relief, much in the same manner as fomentations of warm water which had been previously employed.

Various other remedies were used, but without effect. Opiates afforded but little ease and increased the constipation. Natron and kali were repeatedly had recourse to without deriving any advantage from them. The sulphuric acid was also taken, which at first appeared to mitigate the violence of the symptoms, but soon lost that effect. The patient was now rapidly sinking under the violence of the disease. I therefore, as a last resource, determined to affect the mouth by mercury, particularly as I had experienced so much advantage from this plan in various other diseases. The disorder had now continued with unabated violence upwards of four weeks, and as the patient was reduced to extreme debility, it was necessary to proceed with great caution lest the mouth might be too much affected.

He began by taking two grains of calomel night and morning. On the fourth day after, he complained of a brassy taste in his mouth, and his teeth were somewhat loose. Next day the fœtor of his breath was very evident, and some soreness of his mouth was perceptible. He felt himself easier and had some hours rest in the night. The morning pill was now omitted, and that in the evening taken as usual. His mouth continued to be slightly affected for some days, and all the symptoms rapidly declining, when suddenly the mercurial affection disappeared, and the disease returned with more violence than before.

The quantity of calomel was now increased, and the mouth became again affected, to the great relief of the patient, which unfortunately was but of short duration, as the mercurial action again suddenly subsided, and the original disease returned as before. This alternation of two diseased actions occurred no less than six times, although he was taking 12 grains of calomel daily. So sensible was he of the affection of his mouth relieving him from his other distressing complaint, that he earnestly begged something more might be done to keep his mouth sore. In order to prevent another relapse, and to keep up a steady mercurial action, a drachm of the ung. hydrarg. fort. was rubbed into the legs every night and morning, and six grains of calomel taken in the course of the day. In a few days the tongue and inside of the mouth became ulcerated, but not to that degree as to prove the least impediment to his speech, and a considerable flow of saliva took place.

From this time the symptoms disappeared entirely; his bowels acted regularly without assistance, and his stools became of a natural colour. The affection from the medicine was kept up for three weeks, when he could sit up and considered himself as recovering his strength, although he could not indulge a craving appetite for food, an effect which is very common from the mercurial disease.*

His convalescence proceeded satisfactorily till the 14th of August, when being much disturbed by a domestic quarrel, he went out during a heavy shower of rain, and afterwards remained several hours in his wet clothes. Next day he complained of great stiffness of his joints, with shooting pains in various parts, which soon increased to a most excruciating degree; particularly in the extremities and larger joints, accompanied with much soreness but no tumefaction. His case now became particularly distressing. He had no sleep, and from a perpetual restlessness he required to be frequently

* In all cases in which I have had recourse to this mode of cure, the specific effects of the remedy have been pushed till the symptoms of the disease disappeared, *but no farther;* and the mouth kept in this state from one week to three according to the exigency of the case.

moved, moved, which tended to aggravate his sufferings on account of the extreme tenderness of every part. Various remedies were employed with little or no advantage. When immersed to the neck in warm water he became easy, but a few minutes after the pains returned more violent than before. At one time he lay several hours apparently in a dying state: the whole body was covered with a cold clammy sweat, the eyes half closed, the pulse gone at the wrist, and respiration scarcely to be perceived. He entirely lost the use of his limbs, and the little aliment he took was put into his mouth by those about him. That his pains would cease upon reproducing the mercurial disease, I had not a doubt, but at the same time it seemed not improbable that, in his exhausted state, a second salivation would prove fatal. He, however, took a considerable quantity of the medicine without any effect whatever being produced. At last I was induced to make trial of the nitric acid, and lay aside every other medicine. Accordingly, in the latter end of October, he began taking a drachm of the acid, properly diluted, in the course of the day. His pains began to abate, and he had some sleep at intervals, and before he had taken the medicine eight days he was nearly free from pain, and had a tolerable appetite.

The acid, in the same doses, was continued regularly, without inconvenience, upwards of a month, when he was again in a state of convalescence. His pains had ceased entirely for some time; he had recovered the use of his limbs, and enjoyed sound sleep with an excellent appetite. I now entertained a hope of his complete recovery, but in a very short space of time had the mortification to see all the symptoms of his original disorder again stealing gradually upon him. Every endeavour was used to arrest its progress, but in vain; the disease daily gained ground, and at last exceeded in violence the former attack. In this hopeless situation I considered the patient as fast approaching the end of all his sufferings; yet unwilling to leave him entirely to his fate, I thought of making trial of allum in repeated doses, though with very little hope of success. On the 10th of January last he began taking fifteen grains of common allum, with three grains of nutmeg, in a little mint tea, every six hours. After taking four doses his pains had increased, and on the supposition that the powders were the cause, he was unwilling to persevere. With some persuasion he again began their use, and in the course of twelve hours the pains were much relieved. The bowels now began to act without assistance, and the fœces, which heretofore had been of a dark colour, had had now a reddish hue, an appearance which I have always observed when allum is taken in any quantity.

The powders were regularly taken every six hours for fourteen days, and after that period twice a day for a fortnight longer. As he was then free from complaint, they were omitted. Since that time he has had no occasion for any medicine whatever. He has now much recovered his strength, his appetite is good, he sleeps well, and is about to return to his usual employment.

### REMARKS.

Although there is nothing remarkable in the symptoms of this case, yet it affords an instance of the long continuance of diseased action, where neither fever nor inflammation are present, without producing organic affection.

The Editors of the Medical Journal favoured me with the insertion of a letter in the 21st Vol. on the Hunterian doctrine, that "no two actions can take place in the same constitution nor in the same part at one and the same time." Besides the facts there adduced, nine cases are recorded illustrative of this doctrine, the present case is, however, a much stronger proof of its being founded in truth: In it we see the artificial diseased action, at first, only suspend the original, and the latter, as if having acquired additional strength, by suspension, over-power the former and cause it to disappear. Again, when the ptyalism is renewed, the former disease is suspended a second time, but returns, and the other disappears; and thus continue to alternate for six times. In what way can these phenomena be accounted for, if not on the principle of Mr. Hunter?

Drs. Warren and Biss relate their success in the cure of Colica Pictonum by means of a salivation, and observe *that as soon as the ptyalism was perceived the pain abated and returned no more.* Since that time the power of mercury in subduing the disease has been repeatedly noticed. If mercury possessed any other power over this disorder than that of simply producing a contrary action, why was it not evident, in some degree, in the present case? Although the system was constantly charged with it, yet the instant its effect on the mouth disappeared, the disease returned; or, in other words, the mercurial action proved repeatedly too feeble to resist that of the disease. This mode of curing one disease by inducing another, which we, in the generality of cases, can command, is by no means confined to colica pictonum, or any other disease individually, but has been extended to a great variety with success; a few instances of which may be seen in the cases already alluded to, in the twenty-first volume, and many more might be added.

Mr.

Mr. Hunter's principle has been long generally known; yet, if we may judge from the writings of practical authors, it is seldom acted upon in the cure of diseases.

In many late publications, and in several numbers of the Medical Journal, we find cases where a ptyalism has *unintentionally* been brought on while using some preparation of mercury, and in which the authors mention the fact in a manner that plainly shows they were not prepared to expect the cure of the disease from such an effect.

As this is not the proper place to enlarge on a subject so comprehensive, I shall only recommend to practitioners from my own experience, to keep in view the Hunterian principle, whether they direct their efforts by the use of mercury or by any other means.

With respect to the pains experienced by this patient from which he suffered so severely, it may be supposed they were the natural consequence of the disease, similar to those pains and paralytic affection in which such disorders generally terminate. This was certainly not the fact, his complaint at this time bore no resemblance to those affections, and are to be attributed entirely to his exposure to wet, before the mercurial influence had subsided. It is well known that in such circumstances the only remedy which has been found effectual, is reproducing the ptyalism. In the present case, however, the use of the nitric acid was productive of the best and most speedy effects, and it is not to be doubted but it may, in similar cases, supersede the disagreeable necessity of again subjecting the patient to a salivation.

The last attack of the disorder was very unexpected after so long an interval: it probably would have returned sooner had not the painful affection of the muscles and articulations been present, as it made its appearance after only four days convalescence. To have subjected him to a second salivation in the debilitated state he then was, would have been in all probability attended with fatal consequences. The exhibition of the allum, however, happily superseded the necessity of having recourse to that or any other hazardous means. This remedy seems not to be so generally employed in practice as it deserves. In Drew's case it was productive of the best effects, and in many others which have come under my observation, when it could be retained on the stomach, which is not always the case Dr. Grashuis commends it as a specific in this disorder, and Dr. Percival relates the success which has attended its use in various painful disorders of the bowels.

What is the modus operandi of allum, in such cases?—

Does

Does this drug, and the cuprum vitriolatum, so successfully employed in the West Indies, prove effectual by their astringent powers?

